# Increasing upper limb training intensity in chronic stroke using embodied virtual reality: a pilot study

**DOI:** 10.1186/s12984-017-0328-9

**Published:** 2017-11-17

**Authors:** Daniel Perez-Marcos, Odile Chevalley, Thomas Schmidlin, Gangadhar Garipelli, Andrea Serino, Philippe Vuadens, Tej Tadi, Olaf Blanke, José d. R. Millán

**Affiliations:** 1 MindMaze SA, Lausanne, Switzerland; 2 Laboratory of Cognitive Neuroscience, Brain-Mind Institute, Ecole Polytechnique Fédérale de Lausanne, Lausanne, Switzerland; 3 Center for Neuroprosthetics, Ecole Polytechnique Fédérale de Lausanne, Lausanne, Switzerland; 4 Department of Clinical Neurosciences, University Hospital of Lausanne, Lausanne, Switzerland; 5 Clinique Romande de Réadaptation, Sion, Switzerland; 6 Chair in Brain-Machine Interface, Ecole Polytechnique Fédérale de Lausanne, Lausanne, Switzerland

**Keywords:** Stroke, Neurorehabilitation, Virtual reality, Rehabilitation dose, Motor rehabilitation, Training intensity, Embodied feedback

## Abstract

**Background:**

Technology-mediated neurorehabilitation is suggested to enhance training intensity and therefore functional gains. Here, we used a novel virtual reality (VR) system for task-specific upper extremity training after stroke. The system offers interactive exercises integrating motor priming techniques and embodied visuomotor feedback. In this pilot study, we examined (i) rehabilitation dose and training intensity, (ii) functional improvements, and (iii) safety and tolerance when exposed to intensive VR rehabilitation.

**Methods:**

Ten outpatient stroke survivors with chronic (>6 months) upper extremity paresis participated in a ten-session VR-based upper limb rehabilitation program (2 sessions/week).

**Results:**

All participants completed all sessions of the treatment. In total, they received a median of 403 min of upper limb therapy, with 290 min of effective training. Within that time, participants performed a median of 4713 goal-directed movements. Importantly, training intensity increased progressively across sessions from 13.2 to 17.3 movements per minute. Clinical measures show that despite being in the chronic phase, where recovery potential is thought to be limited, participants showed a median improvement rate of 5.3% in motor function (Fugl-Meyer Assessment for Upper Extremity; FMA-UE) post intervention compared to baseline, and of 15.4% at one-month follow-up. For three of them, this improvement was clinically significant. A significant improvement in shoulder active range of motion (AROM) was also observed at follow-up. Participants reported very low levels of pain, stress and fatigue following each session of training, indicating that the intensive VR intervention was well tolerated. No severe adverse events were reported. All participants expressed their interest in continuing the intervention at the hospital or even at home, suggesting high levels of adherence and motivation for the provided intervention.

**Conclusions:**

This pilot study showed how a dedicated VR system could deliver high rehabilitation doses and, importantly, intensive training in chronic stroke survivors. FMA-UE and AROM results suggest that task-specific VR training may be beneficial for further functional recovery both in the chronic stage of stroke. Longitudinal studies with higher doses and sample sizes are required to confirm the therapy effectiveness.

**Trial registration:**

This trial was retrospectively registered at ClinicalTrials.gov database (registration number NCT03094650) on 14 March 2017.

## Background

Stroke affects about 17 million people per year worldwide, with an increasing rate every year [1]. Stroke survivors often suffer from physical and mental disabilities, heavily impacting their quality of life. Five years after the first stroke, nearly 66% of patients exhibit different degrees of disability and only 34% are functionally independent in their activities of daily living [2].

### Motor rehabilitation after stroke

Motor dysfunction is the most prevalent impairment, with 9 out of 10 stroke survivors suffering from some form of upper limb motor disability [3], and it is a strong predictor of poor functional recovery [4]. Thus, there is a strong need for rehabilitative approaches enhancing motor recovery for stroke patients [5]. To maximize neural, motor and functional recovery, training needs to be long-lasting, challenging, repetitive, task-specific, motivating, salient, and intensive [6]. Standard motor rehabilitation after stroke typically includes neurofacilitation techniques, task-specific training and task-oriented training [7]. Further approaches include strength training, trunk restraint, somatosensory training, constraint-induced movement therapy, bilateral arm training, coordination of reach to grasp, mirror training, action observation and neuromuscular electrical stimulation [8].

Time scheduled for therapy and its frequency are determinant factors for the outcome of motor rehabilitation [9], with a recommended initial amount of at least 45 min for a minimum of 5 days per week [10]. However, the frequency of the delivered therapy usually decreases with time, with therapy being discontinued between 3 and 6 months after the vascular accident [7]. Under these rehabilitation conditions, recovery of motor function has been observed to be strongest during the first month after stroke and to slow down during subsequent months, reaching a “plateau” by 3–6 months post stroke [11, 12]. Clinical evidence for motor improvement in chronic stroke [13] suggests that the “plateau” may depend not only on neurobiological factors, but may also be caused by other factors such as reduction in rehabilitation services [14].

Thus, increasing therapy dose, also in the chronic phase of the disease, might be a critical factor to achieve a positive outcome. Although several guidelines for upper limb rehabilitation have been recently issued [5, 10], the relationship between training intensity and recovery patterns is not yet fully established. Indeed, it is not fully clear how to quantify the dose increase leading to a positive outcome. Training volume, understood as the number of repetitions, seems to be a more relevant parameter of dose than just the total time allocated for therapy [9]. An important issue is how to quantify and capture this concept in a measurable parameter. Intensity of training, understood as the number of repetitions divided by the number of minutes of active therapy, might be a fundamental factor (together with amount and frequency of therapy) to quantify training efficiency. This knowledge becomes critical in order to evaluate cost-effectiveness of new technology-mediated interventions and to select the most valuable therapy procedures at the different stages of the continuum of care for stroke survivors.

### Virtual reality for motor rehabilitation

Different complementary solutions have been proposed during the last decades to help increase and maintain the rehabilitation dose in the long term, mainly through continued therapy. Virtual reality (VR) based motor rehabilitation is a relatively recent approach, showing evidence of moderate effectiveness in improving upper limb and ADL function when compared to conventional therapy [15].

Many VR setups, and often generic (i.e. not developed for rehabilitation purposes) commercial off-the-shelf computer games, are used to perform a series of exercises, where patients move in front of a console and receive mostly visual feedback about their movements [16–18]. This represents a limited approach, whereby the level of immersion and potential feedback is restricted to a single sensorimotor action-perception loop: the patient moves and receives only abstract visual feedback from the screen. A rather different approach implies embodied sensorimotor feedback, where movements of the patient in the real world are reproduced as movements of an anthropomorphic avatar in the virtual environment. Under such conditions, VR allows for more elaborated sensorimotor activation, which may impact the recovery process. In particular, through sensorimotor resonance mechanisms, embodied sensorimotor feedback allows the integration of motor priming techniques and cognitive principles related to body perception and action, including mirror therapy [19] and action observation [20, 21], which have been shown to improve functional recovery and increase cortical activation of the ipsilesional side after stroke. This embodied technology can be achieved by using motion capture technology that interprets the patient’s movements and provides multisensory (vision, audio, touch) feedback to the user about the movement performance. Such enriched VR experiences have been demonstrated to increase patients’ motivation [22] and facilitate functional recovery by engaging appropriate neural circuits in the motor system [23].

One of the VR advantages is that it enables simulated practice of functional tasks at a higher dosage than traditional therapies [15]. Lohse and colleagues recently reviewed the duration, time and frequency scheduled for different VR and computer games interventions, but training intensity (as defined above) was no reported [24]. In general, authors reported an overall median of 570 min of VR (or computer games) therapy delivered, with duration ranging from 20 to 60 min per session, and 8 to 36 sessions [24]. Otherwise, intensity of training is rarely reported for VR training (see [25] for an exception). However, this is a critical factor to estimate cost-effectiveness of VR-based interventions.

### Objectives of the study

The present study aims at investigating the feasibility of admninistering intensive training in chronic stroke patients using a dedicated VR-based system that embeds real-time 3D motion capture and embodied visual feedback to deliver functional exercises designed to train impaired motor skills of the upper limb. Our primary goal was to assess (i) rehabilitation dose and training intensity in chronic patients. Additionally, we asked (ii) whether chronic stroke survivors improve functional outcomes of the upper limb when exposed to intensive VR-based therapy, and we measured (iii) safety and tolerance to such a technology-mediated intervention. We hypothesize that intensive VR-based rehabilitation may lead to high rehabilitation doses and functional improvement in chronic stroke survivors.

## Material and methods

### Participants

Ten chronic (>6 months from stroke onset) outpatient stroke survivors with hemiparesis participated in the study (age: 54.9 ± 13.1 years; 6 females; time after stroke: 6 to 108 months; see Table 1). They were recruited from the “Clinique Romande de Réadaptation” (Sion, Switzerland) from February to October 2015. They were selected following the inclusion and exclusion criteria listed in Table 2.

**Table 1:** Demographic data of participants

Patient	Age (years)	Gender	Stroke	Time from stroke (months)
P1	43	F	Right Sylvian ischemic	51
P2	50	M	Left Sylvian ischemic	26
P3	55	F	Right Sylvian ischemic	108
P4	38	F	Left rupture cerebral aneurysm Sylvian	6
P5	64	M	Left Sylvian ischemic	9
P6	64	M	Left pontine ischemic	22
P7	72	M	Right Sylvian sub-cortical ischemic	72
P8	34	F	Left Sylvian ischemic	6
P9	68	F	Right Sylvian ischemic	42
P10	61	F	Right anterior communicating artery ischemic	54

**Table 2:** Inclusion and exclusion criteria

Inclusion criteria	Exclusion criteria
• Ischemic or hemorrhagic minor-to-moderate (0 < NIHSS < 16) stroke with hemiparesis and experiencing arm motor difficulties • At least 6 months after stroke incident • Maximum 4 on the Medical Research Council Scale (MRCS) for shoulder elevation and elbow flexion/extension • 18 years and older • First ever stroke	• Participating in another movement treatment study at the time of the present study • Severe cognitive impairment (Mini Mental Status Examination score < 18 points) • Orthopedic impairment or visual disorders limiting the treatment • Unable to give informed consent form • Risk of epileptic seizures

### Experimental procedure

This was a single-group and blinded-assessor intervention study, with a physical therapist delivering the therapy, and a second therapist carrying out the pre- and post-intervention assessments. The blinded assessor was not aware of the therapy details, including exercise schedule, dose or training intensity. The clinical protocol was approved by the Ethics Committee of the Canton of Valais (Switzerland). All participants provided their written informed consent prior to enrolment. They were reimbursed for their transportation expenses.

The intervention consisted of 10 one-hour training sessions of VR, with a frequency of two sessions per week over a period of 5 weeks. At each session, the participants sat in a comfortable chair with armrests in front of a table with their feet resting on the floor or on a footstool if needed. The VR system was placed on the table, leaving enough workspace for participants to complete the exercises. A physical therapist freely administered the upper limb therapy content using the VR system, selecting the task/exercise and training modality, and gradually adjusting its difficulty level (e.g. asking the participant to carry it out against gravity or gravity-compensated) according to the participant’s needs and abilities, but without assisting participants physically. At each session, participants completed a series of assessments before and after the training period (see section 2.4). Besides the VR sessions, the participants were allowed to continue their usual therapy sessions and activities of daily living.

The assessor evaluated the participants at baseline (T0), post-treatment (T1) and at 4-week follow-up (T2). The baseline assessment was conducted prior to the beginning of the training. The post-treatment assessment was conducted after a 20-min break after the last training session. Follow-up assessments were completed four weeks after the end of the training (i.e., nine weeks after baseline).

### Virtual reality system

**Fig. 1:**
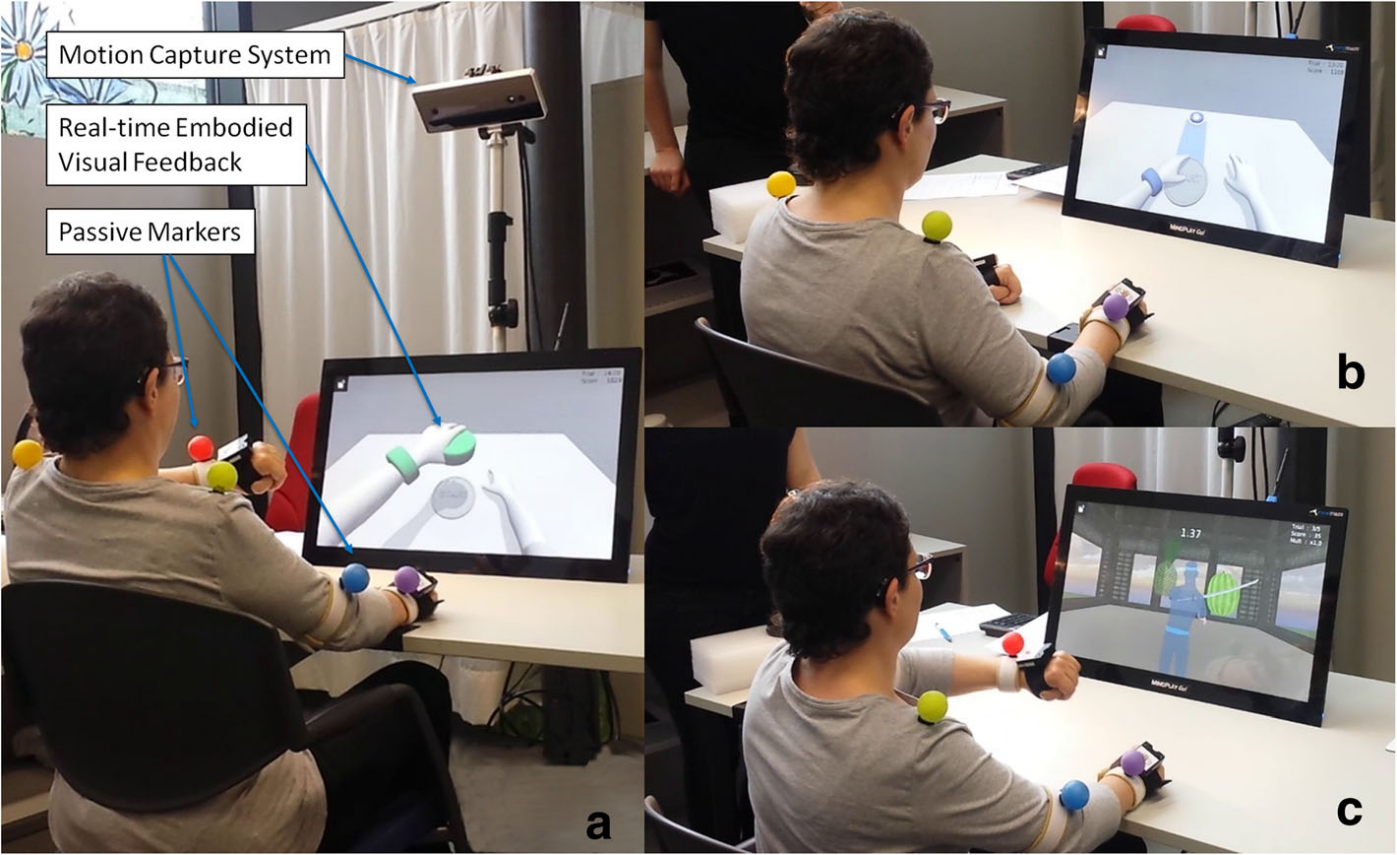
**a** Participant performing an upper limb exercise (Grasping) with the MindMotion ™ PRO technology; **b** Participant doing the Reaching exercise; **c** Participant doing a Fruitchamp exercise

The interventional device was a tabletop version of MindMotion™ PRO (MindMaze SA, Switzerland), a VR-based motor rehabilitation system developed for functional training of upper limb after brain damage. Exercises of the MindMotion™ PRO are presented in game-like scenarios designed to increase patients’ motivation and therapy dose. The mobile platform is composed of a 3D motion tracking camera (MindMaze SA), and a touch screen with an embedded computer. The 3D motion tracking camera captures and interprets participant’s movements, quantifying upper limb and trunk joints angles by using passive colored markers (Fig. 1a). For tracking of the forearm (supination and pronation) and wrist (flexion-extension, ulnar-radial deviation) movements, wireless inertial trackers using Bluetooth technology are used. These movements are then mapped to an avatar (i.e., a virtual character) in the virtual environment. The avatar, seen from a first-person or third-person perspective, reproduces the participant’s movements in real time (Fig. 1), ensuring visuomotor synchrony and closed-loop via embodied visual feedback (participant identifies his/her own movements in those of the avatar). The touch screen includes a button that allows the user to switch between the therapist interface, where therapist composes and launches the exercises, and the patient interface, which displays the virtual environment for the VR exercises. Through the user interface, therapist prescribes the appropriate exercises by selecting them from the available set, indicating the body side to be used, the visual feedback modality, difficulty level and number of repetitions. The device’s database stores all information related to therapy execution, therefore allowing for accurate quantification of training intensity.

#### The rehabilitation exercises

The system offers interactive VR exercises that engage participants' shoulder, elbow, forearm and wrist movements with various levels of difficulty. These movements are integrated into functional tasks that include pointing, reaching and grasping virtual objects. The pointing exercise consists of aiming at the center of a target with the forearm or wrist during a few seconds. In the reaching task (Fig. 1b), the participant has to extend the arm to hit virtual objects placed on a virtual table within the peripersonal space. The grasping game consists of grabbing a virtual object in the vertical plane (illustrated in Fig. 1a) and dropping it in a new location. The most solicited joint movements include: shoulder flexion/extension, shoulder horizontal abduction/adduction, shoulder internal/external rotation, and elbow flexion/extension. Each exercise includes a variant to additionally train forearm pronation/supination and wrist flexion/extension. All exercises are played from a first-person perspective and can be done with participants sliding their arms on the table surface, to help to compensate for gravity. When needed, therapists offered to use a small towel to ease sliding of the arm over the table surface. These tasks aim at improving movement stability, trajectory accuracy, and muscle strength against gravity. An additional exercise in an enriched scenario, where a ninja seen from a third-person perspective cuts fruits appearing on the screen, develops participants' range of motion and motor control while increasing speed and accuracy of movements of the forearm and wrist (Fig. 1c). After each repetition, participants receive a score based on their motor performance (i.e., movement stability, trajectory accuracy) as a reward for enhancing their motivation.

The system also includes a virtual mirror mode available for all exercises, where movements of the unaffected arm control the movements of the contralateral virtual arm, providing the visuomotor illusion of movement of the affected arm. Therapists were encouraged to promote activity of the affected arm using the direct mode, however they could freely select the mirror mode when appropriate, e.g. for the affected arm to get some rest if fatigue appears. Additionally, and in line with the concept of constraint-induced movement training that forces use of the affected hand [26], the virtual exercises with MindMotion™ PRO software allow to select the targeted arm (left/right) for the game control, so that the participant can progress only if the targeted limb is used.

### Outcome measures

#### Primary outcomes

##### Rehabilitation dose and training intensity

Rehabilitation dose and training intensity were quantified according to the following variables:

i. Duration of the training session, defined as the number of minutes from the beginning of the first exercise of the session to the end of the last exercise of the session.
ii. Effective training time, defined as the number of minutes a participant actively trained during each session. Breaks between exercises were excluded from this measure.
iii. Number of goal-directed movements, defined as the sum of intended movements (e.g. elbow extension to reach, shoulder internal rotation to point, elbow flexion to come back to initial position) to achieve a task, per session and in total.
iv. Number of goal-directed movements per minute of effective training time.

Importantly, the total number of goal-directed movements reflects the overall rehabilitation dose. The number of goal-directed movements per minute of effective training time yields an estimate of the intensity of the training. All the recorded times were extracted from the database of the VR system.

#### Secondary outcomes

##### Upper limb function

Upper limb function was assessed with the Fugl-Meyer Assessment for Upper Extremity (FMA-UE), a stroke-specific test to measure motor impairment and determine motor recovery [27]. In chronic stroke, the minimal clinically important difference (MCID) for the overall upper limb function measured with FMA-UE is 5.25 over a maximum score of 66 [28]. FMA-UE was administered at baseline (T0), after completion of the last treatment session (T1), and at follow-up one month after completion of the training (T2).

##### Active range of motion

Active range of motion (AROM) of shoulder flexion (0° position: arm by side aligned between shoulder and hip), elbow extension (0° position: fully extended elbow; humerus and radius aligned), wrist extension (0° position: hand resting on table with palm facing down), forearm supination (0° position: thumb oriented up towards ceiling) and pronation (0° position: thumb oriented up towards ceiling) was measured with a goniometer in a standardized way (neutral zero method) [29]. This measure evaluates participant’s capacity to perform isolated joint movements.

##### Muscle strength

Muscle strength for each joint was assessed with the Modified Medical Research Council Scale (mMRCS) [30].

##### Functional independence

Functional independence was assessed with the Functional Independence Measure (FIM; maximal score: 126), which evaluates motor function and socio-cognitive skills [31].

##### Pain ratings

In addition to the questions related to adverse events, we measured pain level at each joint (shoulder, elbow, wrist) at the beginning of each session using an 11-point visual analog scale (VAS) [32].

AROM, mMRCS, FIM and pain VAS were administered at baseline (T0), after completion of the last treatment session (T1), and during a follow-up visit one month after completion of the training (T2).

#### Safety and acceptance of technology

Before and at the end of each session, participants answered 15 questions that evaluate different aspects on safety and acceptance of the technology:

##### Tolerance to VR intervention

Q1-Q4 were related to fatigue and relaxation, comparing participant’s states immediately before and after each session (Table 6). This comparison provided us information whether therapy sessions increased fatigue and stress levels. This information was recorded at every session.

##### Adverse event monitoring

Q5 referred to any pain experience during the training (Table 6). This information was recorded at every session. Besides questionnaire, participants were debriefed at the end of each session.

##### Self-evaluation

Q6 assessed self-reported movement improvements (Table 6). This information was recorded at every session.

##### Acceptance of technology

Q7-Q8 evaluated the degree of concentration and immersion into the VR exercises. Q9 reported on the motion tracking accuracy. Q10-Q13 reflected participant’s attitude towards the technology. This information was recorded at first and last sessions (Table 7).

##### Motivation

Q14-Q15 referred to participant’s motivation to continue the therapy at the hospital and at home (Table 7). This information was recorded at first and last sessions.

To quantify participant’s responses, we used a 7-point colored visual scale, with participants pointing to the level that corresponded best to their state, which was translated into a 7-point ordinal scale (1 = completely disagree, 7 = completely agree). After the questionnaire, participants had the chance to give any additional feedback regarding the exercises and treatment.

### Data analysis

Data analysis was conducted with *R* software [33]. We used Wilcoxon signed-rank test for the single comparisons (last vs. first session) of the rehabilitation dose and training intensity measures. We applied non-parametric Friedman test to evaluate changes in the secondary outcome measures from baseline (T0) to post-intervention (T1) and follow-up (T2). We also used Wilcoxon signed-rank test for the post-hoc analyses, and reported effect sizes (r) [34]. Improvement rate in FMA-UE was computed as the improvement percentage with respect to the potential full recovery (i.e. 66-baseline score). Non-parametric Friedman test was also conducted to estimate any changes in fatigue and relaxation levels within sessions across the treatment. For all the analyses, we set significance level at *p* < 0.05, and then applied Bonferroni correction when multiple comparisons were made. We report Median and Interquartile Range (IQR) unless otherwise specified.

## Results

All ten participants completed all ten sessions of the treatment. The study lasted nine months and one week, counted from the first session of the first participant to the follow-up session of the last patient. No severe adverse events were reported. Overall, 1485 tasks were completed (Point: 20.5%; Reach: 41.3%; Grasp: 23.4%; Fruitchamp: 14.9%); only 1.35% used the mirror mode.

### Primary outcomes

#### Rehabilitation dose and training intensity

Table 3 summarizes all analyzed components of the rehabilitation dose. The median duration across participants of the training sessions increased from 26.8 min (IQR: 20.6 to 32.7) in the first session to 37.2 min in the last session (IQR: 30.9 to 45.6; non-significant trend, Z = 1.687, *p* = 0.074, effect size *r* = 0.377). The total training time across all ten sessions was 403 min (IQR: 331 to 417). More importantly, the median effective training time per session increased from 16.5 min (IQR: 12.5 to 20.1) in the first session to 32.1 min in the last session (IQR: 23.9 to 37.9; Z = 2.701, *p* = 0.007, *r* = 0.604). The total effective training time provided across the ten sessions was 290 min (IQR: 246 to 329).

**Table 3:** Differences in the rehabilitation dose between the first and last sessions. * Wilcoxon signed-rank test, *p* < 0.05

	Session #1 Median (IQR)	Session #10 Median (IQR)	Total Median (IQR)	*p*-value (Session #1 vs. #10)
Duration of training (minutes)	26.8 (20.6 to 32.7)	37.2 (30.9 to 45.6)	403 (331 to 417)	0.074
Effective training time (minutes)	16.5 (12.5 to 20.1)	32.1 (23.9 to 37.9)	290 (246 to 329)	0.007*
Goal-directed Movements	212.0 (152.0 to 301.3)	476.5 (432.3 to 637.0)	4713 (3669 to 5293)	0.005*
Goal-directed movements per minute of Effective training time	13.2 (11.4 to 15.9)	17.3 (16.6 to 18.7)	N/A	0.037*

**Fig. 2:**
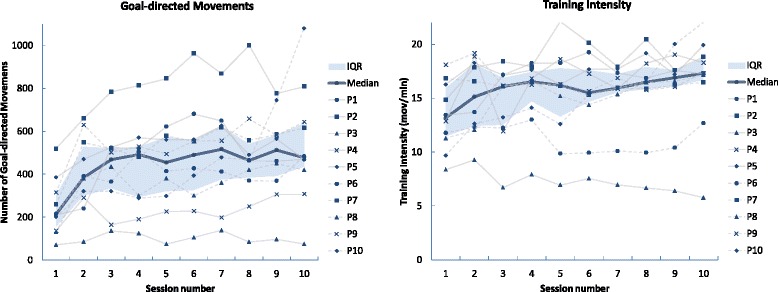
Individual, median and IQR values for Goal-directed Movements (left) and Training Intensity (right) at each session

The median number of goal-directed movements per session increased from 212.0 (IQR: 152.0 to 301.3) in the first session to 476.5 in the last session (IQR: 432.3 to 637.0; Z = 2.805, *p* = 0.005, *r* = 0.627), with a maximum of 517.0 (IQR: 373.0 to 624.3) in session #7 (Fig. 2). The total of goal-directed movements completed by patients across the ten sessions was 4713 (IQR: 3669 to 5293).

Notably, the intensity of the training, defined as the number of goal-directed movements per minute of effective training time, increased progressively from 13.2 (IQR: 11.4 to 15.9) in the first session to 17.3 movements in the last session (IQR: 16.6 to 18.7; Z = 2.089, *p* = 0.037, *r* = 0.467; Fig. 2).

### Secondary outcomes

#### Upper limb function

Changes in FMA-UE scores were observed across the different assessment time points (χ^2^(2) = 9.892, *p* = 0.007)**.** When performing a post-hoc analysis, FMA-UE score increased from 42.0 (IQR 24.75 to 53.0) at T0 (baseline) to 44.5 at T1 (IQR: 26.25 to 54.75; Z = 1.552, *p* = 0.131, effect size *r* = 0.347) and increased to 45.5 at T2 (IQR: 27.0 to 57.0; Z = 2.105, *p* = 0.035, *r* = 0.471; Table 5). Participants P2 and P4 improved in FMA-UE more than the MCID at post-treatment and follow-up (P2: 47➔54➔54; P4: 17➔24➔26). Participant P10 also improved over the MCID at follow-up (54➔55➔61). Table 4 shows FMA-UE scores for each participant. Studies suggest that motor recovery is better captured in terms of change in functional scores, rather than of final endpoint [35]. Thus, we also analyzed the proportion of recovery obtained after the training and the follow-up, by calculating the score difference normalized for the maximum recovery possible. This measure better controls for individual differences at baseline and possible ceiling effect. After the VR-based intervention, the median improvement rate was of 5.3% from T0 to T1, and of 15.4% from T0 to T2. In both cases, the improvement was significantly greater than zero (Wilcoxon signed-rank test; T0-T1: *p* = 0.038; T0-T2: *p* = 0.014).

**Table 4:** FMA-UE and improvement rates for each participant at baseline (T0), post-assessment (T1) and follow-up (T2)

FMA-UE	P1	P2	P3	P4	P5	P6	P7	P8	P9	P10
Baseline (T0)	36	47	55	17	21	37	57	18	50	54
Post-treatment (T1)	33	54	55	24	23	38	58	17	51	55
Follow-up (T2)	30	54	58	26	23	39	59	19	52	61
Improvement rate at T1	−10%	37%	0%	14%	4%	3%	11%	−2%	6%	8%
Improvement rate at T2	−20%	37%	27%	18%	4%	7%	22%	2%	13%	58%

#### Active range of motion

A significant pre-post improvement in AROM was observed for shoulder flexion (χ^2^(2) = 9.297, *p* = 0.010), likely the joint movement solicited the most in the ensemble of VR exercises. Shoulder flexion increased from 105.0° (IQR: 63.8° to 118.8°) at T0 (baseline) to 117.5° (IQR: 71.3° to 133.8°; Z = 1.843, *p* = 0.065, effect size *r* = 0.412) at T1 (post-treatment) and significantly to 117.5° (IQR: 77.5° to 131.3°; Z = 2.770, *p* = 0.007, *r* = 0.619) at T2 (follow-up). A positive change was also observed for forearm pronation (χ^2^(2) = 6.889, *p* = 0.032). Forearm pronation increased from 62.5° (IQR: 0.0° to 83.8°) at T0 to 87.5° (IQR: 56.3° to 90.0°; Z = 2.390, *p* = 0.027, *r* = 0.534) at T1 and to 87.5° (IQR: 57.5° to 90.0°; Z = 1.714, *p* = 0.128, *r* = 0.383) at T2. No significant changes in AROM were found for the rest of upper limb movements measured (Table 5).

**Table 5:** Median and IQR of primary and secondary clinical outcomes at baseline (T0), post-treatment (T1) and follow-up (T2). ^+^ Friedman test, *p* < 0.05; * Wilcoxon signed-rank test, *p* < 0.025 (Bonferroni corrected)

	Baseline (T0) Median (IQR)	Post-treatment (T1) Median (IQR)	Follow-up (T2) Median (IQR)	Friedman test	Wilcoxon signed-rank test	
				p-value	p-value T0 to T1	p-value T0 to T2
FMA-UE	42.0 (24.75 to 53.00)	44.5 (26.25 to 54.75)	45.5 (27.0 to 57.0)	0.007^+^	0.131	0.035
AROM (in °)						
Shoulder flexion	105.0 (63.8 to 118.8)	117.5 (71.3 to 133.8)	117.5 (77.5 to 131.3)	0.010^+^	0.065	0.007*
Elbow extension	−8.5 (−10.0 to −1.3)	−7.5 (−10.0 to 0.0)	−5.0 (−10 to 0)	0.420		
Wrist extension	35.0 (6.3 to 55.0)	37.5 (8.8 to 55.0)	45.0 (22.5 to 60.0)	0.095		
Forearm supination	62.5 (5.0 to 78.8)	57.5 (12.5 to 83.8)	67.5 (12.5 to 80.0)	0.131		
Forearm pronation	62.5 (0.0 to 83.8)	87.5 (56.3 to 90.0)	87.5 (57.5 to 90.0)	0.032^+^	0.027	0.128
mMRCS						
Shoulder flexion	3 (3.0 to 3.8)	3.5 (3.0 to 4.0)	3.5 (3.0 to 4.0)	0.156		
Elbow extension	2.5 (2.0 to 3.0)	3 (2.3 to 3.8)	3 (2.3 to 4.0)	0.074		
Wrist extension	3 (1.5 to 4.0)	3 (2.3 to 4.0)	3.5 (2.3 to 4.0)	0.174		
Forearm supination	3 (1.3 to 4.0)	3.5 (1.5 to 4.0)	3.5 (1.5 to 4.0)	0.424		
Forearm pronation	3 (0.5 to 4.0)	3.5 (2.3 to 4.0)	3.5 (3.0 to 4.0)	0.350		
FIM	121.5 (106.3 to 125.3)	121 (105 to 123)	121.5 (108.8 to 123.8)	1		
VAS						
Shoulder flexion	0 (0 to 6)	1 (0 to 2)	0 (0 to 1.75)	0.368		
Elbow extension	0 (0 to 4)	0 (0 to 0)	0 (0 to 0)	0.146		
Wrist extension	0 (0 to 4)	0 (0 to 0)	0 (0 to 0)	0.0498^+^	0.103	0.103
Forearm supination	0 (0 to 0)	0 (0 to 0)	0 (0 to 0)	0.135		
Forearm pronation	0 (0 to 0)	0 (0 to 0)	0 (0 to 0)	0.368		

#### Muscle strength

Strength for each muscle group assessed (i.e., shoulder flexion, elbow extension, wrist extension, forearm supination, forearm pronation) showed an improvement of 0.5 points in mMRCS at follow-up compared to baseline (Table 5). These changes were not statistically significant (*p* > 0.05).

#### Functional independence

Participants showed high levels of function both before and after the treatment. Consequently, FIM scores showed no change from baseline (121.5; IQR: 106.3 to 125.3) to post-treatment (121; IQR: 105 to 123) and to follow-up assessments (121.5; IQR: 108.8 to 123.8; χ^2^(2) = 0, *p* = 1; Table 5). No changes were observed for the six “self-care” items, which are sensitive to changes in paretic upper limb function, between baseline (41; IQR: 37.5 to 42), post-treatment (41; IQR: 37 to 42) and follow-up assessments (41.5; IQR: 38.3 to 42; χ^2^(2) = 4, *p* = 0.135).

#### Pain ratings

Overall, pain levels as measured by the VAS scale at baseline, post-treatment, and follow-up were low all across the treatment, confirming no negative long-lasting effects due to the VR-based intervention. Importantly, pain levels did not increase during the treatment period for any joint (all p > 0.05; Table 5), meaning that physical activity carried out during VR intervention was well tolerated.

#### Relationship between motor improvement and training intensity

**Fig. 3:**
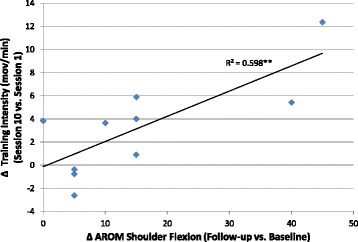
Scatter plot and linear regression between changes in training intensity (session 10 vs. session 1) and changes in AROM of shoulder flexion scores (follow-up vs. baseline). ** Wilcoxon signed-rank test, *p* < 0.01

We explored any eventual relationship between the rehabilitation dose or training intensity and the clinical outcomes capturing any significant change after the intervention, namely FMA-UE and AROM of shoulder flexion. We could not establish any relationship between the total active therapy time with either pre-post changes in FMA-UE (R^2^ = 0.011) or AROM of shoulder flexion (R^2^ = 0.027). The total dose of goal-directed movements could not explain the changes either in FMA-UE (R^2^ = 0.058) or in AROM of shoulder flexion (R^2^ = 0.017). Similarly, an examination of the median training intensity achieved by each patient did not appear to be able to predict changes in FMA-UE (R^2^ = 0.115) and AROM of shoulder flexion (R^2^ = 0.016). Importantly, however, the increase in training intensity observed between the first and last sessions partially explained the changes in AROM of shoulder flexion (R^2^ = 0.187, *p* = 0.212), a relationship that became stronger and significant at the follow-up assessment (R^2^ = 0.598, *p* = 0.009; Fig. 3). Thus, the more patients increased their training across sections, the better the functional outcome. This relationship was specific for shoulder flexion, i.e. the targeted joint in most of the VR activities, but it was not present for general upper limb function, as captured by FMA-UE scores (R^2^ = 0.018).

### Safety and acceptance of technology

#### Tolerance to VR intervention

All participants started every session with similar and low levels of fatigue (median score: 1; IQR: 1 to 3.8; χ^2^(9) = 7.730, *p* = 0.562; Table 6). When comparing the level of fatigue before (Q1) and after (Q3) each session, we found no statistical difference (χ^2^(19) = 18.137, *p* = 0.513). However, the median level of fatigue in session #2 was moderate (median score: 5.5; IQR: 1.5 to 6; Z = 2.545, *p* = 0.01, effect size *r* = 0.569). This occasional increase in fatigue may be related to the considerable increase in the rehabilitation dose from session #1 to session #2, once participants assimilated the technology (see Fig. 2). Regarding relaxation (stress) levels, participants came to each session with similar and high levels of relaxation (median score: 7; IQR: 5.3 to 7; χ^2^(9) = 9.203, *p* = 0.419; Table 6). When comparing the relaxation level before (Q2) and after (Q4) each session, we observed no difference (χ^2^(19) = 25.386, *p* = 0.148), meaning that the intensive VR intervention did not increase participants’ stress.

**Table 6:** Evolution of questionnaire scores related to Safety aspects across sessions

Questions	Session #									
	1	2	3	4	5	6	7	8	9	10
Tolerance to VR intervention										
Q1: Before the session, how tired do you feel?	1 (1 to 3.8)	1 (1 to 2.5)	1.5 (1 to 2.8)	2 (1 to 3)	2 (1 to 4)	1 (1 to 4)	3.5 (1 to 5)	3 (1.5 to 5.8)	1 (1 to 4.5)	1 (1 to 2.5)
Q3: After the session, how tired do you feel?	1 (1 to 5.8)	5.5 (1.5 to 6)	1 (1.5 to 6)	2 (1 to 5)	2 (1 to 5)	2 (1 to 6)	3.5 (1 to 5.8)	3.5 (1.3 to 6)	2.5 (1 to 5.8)	2 (1 to 5.8)
Q2: Before the session, how relaxed do you feel?	7 (5.3 to 7)	7 (7 to 7)	7 (7 to 7)	7 (7 to 7)	7 (7 to 7)	7 (7 to 7)	7 (7 to 7)	7 (7 to 7)	7 (7 to 7)	7 (7 to 7)
Q4: During the session, how relaxed did you feel?	7 (6.3 to 7)	7 (7 to 7)	6 (4 to 7)	7 (6.3 to 7)	7 (7 to 7)	7 (4 to 7)	7 (6.3 to 7)	7 (6 to 7)	7 (5.3 to 7)	7 (6.3 to 7)
Adverse event monitoring										
Q5: During the exercises, did you feel any unusual pain (e.g. stronger) at the level of the upper limbs (arms, joints, hands) or the trunk?	1 (1 to 1.8)	1 (1 to 2.5)	1 (1 to 1)	3 (1 to 5)	1 (1 to 3)	2 (1 to 3)	1.5 (1 to 5)	3.5 (1 to 5.8)	1 (1 to 1.8)	1 (1 to 4.5)
Self-evaluation										
Q6: After the session, do you feel any improvement of you movements (e.g., larger movements, more precise, etc.)?	4 (1.5 to 6.3)	4.5 (4 to 5.8)	6 (4.3 to 7)	6 (3.5 to 7)	6 (5 to 7)	6 (4.5 to 6.8)	5.5 (5 to 6)	6 (4.5 to 7)	6 (4 to 6)	7 (5.3 to 7)

#### Adverse event monitoring

In addition to overall pain ratings at baseline and post-treatment, participants reported any eventual pain felt at the level of the upper limbs and trunk during the training (Q5; Table 6). Self-reported pain was kept low, with no significant changes across sessions (χ^2^(9) = 8.911, *p* = 0.446). Three adverse events were reported. Participant P1 reported increased pain in the shoulder area before the training (from session #7 onwards). Debriefing with the participant and her usual therapist could not reveal the cause of that pain. Participant P4 was administered Botox in upper and lower limbs the day before session #4, which led to higher level of pain in subsequent sessions. Participant P3 was particularly sensitive to screen exposure and needed headache medication. For this patient, the duration of the therapy sessions was reduced to 30 min.

#### Self-evaluation

Participants reported a continuous feeling of improvement on mobility (Q6) across the sessions (χ^2^(9) = 8.037, *p* = 0.530; Table 6). This self-reported improvement significantly increased from 4 (IQR: 1.5 to 6.3) in the first session to 7 (IQR: 5.3 to 7; Z = 2.136, *p* = 0.027, effect size *r* = 0.478) in the last session (Table 7).

**Table 7:** Acceptance of technology, motivation and self-evaluation questions in the first and last sessions. * Wilcoxon signed-rank test, *p* < 0.05

Questions	First Session Median(IQR)	Last Session Median(IQR)	Wilcoxon test *p*-value
Self-evaluation			
Q6: After the session, do you feel any improvement of you movements (e.g., larger movements, more precise, etc.)?	4 (1.5 to 6.3)	7 (5.3 to 7)	0.027*
Acceptance of technology			
Q7: During the exercises, were you concentrated on the task?	7 (7 to 7)	7 (7 to 7)	0.786
Q8: During the exercises, did you have the feeling of being in the hospital room?	1 (1 to 1)	1 (1 to 1.8)	0.103
Q9: Did the movements of the character reflect your movements?	6 (5.3 to 7)	6.5 (5.3 to 7)	0.546
Q10: During the exercises, did you feel comfortable with the requested movements?	5 (4 to 7)	7 (6 to 7)	0.090
Q11: Did you like the exercises?	7 (7 to 7)	7 (7 to 7)	0.317
Q12: Did you have the impression of doing rehabilitation exercises?	7 (4.5 to 7)	7 (3 to 7)	0.706
Q13: Would you like the character to look more realistic?	1 (1 to 4.8)	4 (1.3 to 6.8)	0.438
Motivation			
Q14: Would you like to spend more time doing the exercises at the hospital?	6 (4 to 7)	7 (3.3 to 7)	0.595
Q15: Would you like to continue doing the exercises at home?	7 (4.3 to 7)	7 (5.3 to 7)	1.000

#### Acceptance of technology

From the first session on, participants showed high levels of concentration (Q7; median score: 7, IQR: 7 to 7) and immersion (Q8; median score of feeling at hospital environment: 1, IQR: 1 to 1), even forgetting that they were at the hospital (Table 7). Participants identified to a great extent their movements corresponding to those of the avatar (Q9; median score: 6, IQR: 5.3 to 7), and this perception was maintained across sessions, meaning that the self-identification with avatar’s movements was kept constant (Table 7). Participants liked to a great extent performing the exercises (Q10; median score: 5, IQR: 4 to 7), and were comfortable with the demanded movements (Q11; median score: 7, IQR: 7 to 7) while being aware of the rehabilitative intention of the exercises (Q12; median score: 5, IQR: 4.5 to 7). At the end of the treatment, they also reported some interest in having an improvement in the graphical quality of the avatar (Q13; median score: 4, IQR: 1.3 to 6.8). In all cases, this acceptance of technology was intact after ten sessions of training (Table 7; all *p* > 0.05).

#### Motivation

Participants explicitly expressed their willingness to continue with the VR training both at hospital (Q14; median score after session #1: 6, IQR: 4 to 7) and at home (Q15; median score after session #1: 7, IQR: 4.3 to 7), confirming their adherence to the VR intervention (Table 7). These levels of motivation were maintained intact after ten sessions of training (p > 0.05).

## Discussion

### Primary outcomes

The results of this pilot study on chronic stroke show that dedicated VR-based functional training of the upper limb is able to provide high rehabilitation doses, both in terms of active training time and repetitions per session (i.e., training intensity). Participants received a total of 290 min of active VR-based functional training of the upper extremity, with a total duration of the training sessions of 403 min. The median duration of the training session (including breaks and time between exercises) continuously increased up to 37.2 min in the last session, which approaches the therapy target of rehabilitation guidelines [10]. More importantly, after participants had become familiarized with the system (mostly during the first session), the VR system allowed for very efficient training sessions, with up to 32.1 min of effective physical activity of the upper limb in the last session. The rest of the time was dedicated to the selection of the exercises composing the session and the pauses between exercises. This translates into an efficiency rate (relation between time of therapy session and time spent in active therapy) of 86.3% for the VR-based intervention. This result supports recent evidence proving that VR-based treatments after stroke can be 10% more efficient (i.e., higher activity rate) than conventional therapy (77% vs. 67% of total therapy time), 20% (81% vs. 61%) for severely impaired patients [25].

Animal studies suggest that 400–600 repetitions per day of functional tasks are required to induce structural neurological changes [36]. In our study, participants completed a median of 4713 goal-directed movements (which represented 1834 task repetitions). This translates to an average of 471 movements (183 tasks) completed per session, which represents a quantitatively higher dose as compared to average functional upper extremity repetitions (45 ± 13) provided in outpatient clinical practice [37]. Indeed, recent studies show that the standard dose can be increased with intensive, high-repetition programs [36]. Technology-mediated interventions such as virtual reality [15] and robotic platforms [38] have demonstrated higher dose efficiency. In the present study, VR-mediated training delivered up to 17 goal-directed movements per minute (~8–9 completed tasks per minute), a training intensity 10–15 times higher as in standard therapy [39].

### Secondary outcomes

Participants improved in FMA-UE score, with an overall 15.4% improvement rate at follow-up. Importantly, three patients improved beyond MCID, meaning that the VR-based intervention may have contributed to further clinical improvement even in a late chronic phase. Our results bring further evidence of how highly intensive upper limb training with specific shoulder and arm tasks, delivered by means of a embodied VR system, may help improve AROM for the shoulder flexion in moderate-to-severe chronic stroke patients. This is in line with a recent study that has reported AROM improvements in chronic stroke using VR-based training of moderate intensity (72 repetitions per session), especially in patients with mild upper limb motor deficits [40].

We note that FIM scores did not change after the intervention. This is likely due to the high-level of functional independence already achieved by patients at baseline (median value was 121 out of 126). Another factor could be the fact that residual deficits on chronic patients usually remain for distal and more complex finger movements, which are not targeted by the current VR exercises. Interestingly, a large study involving 376 patients who received 40 h of training in 4 weeks did report an increase in FIM scores for both a VR group and a conventional therapy group, with the VR group improving significantly more than the conventional therapy group [41]. Thus, longer and/or more specific upper limb training may be necessary to elicit positive FIM changes in chronic stroke patients.

We found that the improvements in AROM of shoulder flexion observed after the intervention, and particularly at the follow-up assessment, could be partially explained by the increase in training intensity across sessions, but not by the rehabilitation dose *per se* (neither in number of movements or time spent in the training). This preliminary finding seems to be in line with the recommendations of the recently formed Stroke Recovery and Rehabilitation Roundtable, which advocates that "recovery trials need to consider serially applied kinematic/kinetic measurements alongside clinical assessments to distinguish between restitution and compensation. A core set of kinetics and kinematic outcomes needs to be established" [42]. Within this context, VR systems, in particular motion capture technology, can help quantify changes in motor recovery in an objective fashion. Indeed, kinematic analyses of movement quality (based on high-quality motion tracking recordings) have been strongly advocated to be incorporated into clinical assessments as they may capture better changes in motor control [43, 44].

Embodied technology has been recently proposed for neuroprosthetics [45], and treatment of different pathologies, such as pain management [46–48] or eating disorders [49]. In the present study, we used a novel VR system for motor rehabilitation after stroke that provides embodied visuomotor feedback. In this regard, a randomized clinical trial with stroke patients has shown that VR-mediated embodied feedback for gait training may entrain several brain areas (probably encompassing the mirror neuron system) involved in motor planning and learning, thus leading to an enhanced motor performance [50]. In this context, VR has the unique potential to manipulate visual feedback of the movement made by the participant, in a way which potentially allows the selection of patterns of sensorimotor coherence aimed at activating specific sensorimotor brain circuits (e.g., action observation system [51] and other monitoring systems [52]). The individual contributions of the different priming techniques and cognitive principles included in the provided feedback were beyond the scope of this study and need to be addressed in further studies using dedicated designs.

The results of the present study suggest that it is possible to improve functional skills of the upper limb in stroke survivors with intensive training, even in the early chronic (6 months post stroke) and later chronic phases (54 months post stroke) [13]. However, secondary outcomes did not show clinical improvements after 403 min of training delivered (290 min effective training time). Thus, besides delivery of high training intensity, higher rehabilitation volumes are required to affect FMA and FIM scores in chronic stroke patients who still present functional deficits at everyday life activities. Further studies with stratified groups and different motor assessments could also help to identify (i) the patient profiles that benefit the most from this embodied VR technology, and (ii) the motor outcomes that capture best the eventual improvements.

### Safety and acceptance of technology

Participants reported low levels of fatigue and stress generated during the training sessions. Despite the fact that the rehabilitation dose and intensity increased, especially from session #2 onwards, the level of fatigue did not. No serious adverse event was reported in the present study. This is very likely because the VR exercises were specifically designed for neurorehabilitation purposes by clinicians and physical therapists, and were validated with acute stroke patients before proceeding to this study [53, 54]. This approach differentiates the current embodied VR device from other attempts to adopt computer games for stroke rehabilitation, whereby consumer video consoles are used to provide motor training [17, 18]. Approaches based on the adoption of off-the-shelf, not clinically customized, solutions have serious limitations, in that therapy objectives are not taken into account, with the type and level of activities not being tuned to stroke patients’ residual abilities. In a recent study that used off-the-shelf, non-adapted, videogames for upper extremity training in subacute stroke [17], and with similar training doses delivered, participants reported adverse events such as dizziness (15%), headaches (13%) or nausea (6%).

Motivation and engagement are intrinsic component of VR- and videogame-based interventions in stroke rehabilitation [55]. They are related not only to compliance and adherence to rehabilitation programs, but they also influence intervention outcomes [24]. In the current study, participants showed the highest levels of adherence to the rehabilitation program and motivation to continue therapy both at hospital and at home. This may relate to the very high levels of self-perceived improvement in mobility reported by participants at the end of the treatment. Importantly, during the debriefing several participants reported higher self-confidence levels at activities of daily living at home, with more use of the affected arm, therefore reducing non-learned use effect. This catalyzing effect may have contributed to the observed functional improvements during the follow-up assessment.

### Limitations of the study

Besides the assessment of the primary outcome (training intensity and dose), this pilot study included the collection of several clinical outcomes. Within this regard, the study presents several limitations, the most important being the lack of a control group and a relatively small sample size, which undermine the possibility of providing evidence on therapy effectiveness. The level of physical activity of participants (or inactivity, in particular of the affected limb) prior to the intervention could also not be assessed. This is particularly critical in chronic stroke research, as patients could be functioning at a level below their full potential due to disuse. The duration of the training sessions provided in this study, and consequently the delivered dose, may have been affected by the dependence on the outpatient transportation service, planned for one hour after the session started. Training schedules (twice a week) were also adapted to both outpatient population and physical therapist availability.

## Conclusion

This pilot study has shown the feasibility and safety of a specific and intensive functional training of upper extremity in chronic stroke survivors with a dedicated VR system for neurorehabilitation and based on closed visuomotor loop via embodied visual feedback. The rehabilitation dose was continuously increased, adjusting to patients’ needs to maximize the training efficacy. Functional and motor outcomes suggest that highly intensive VR training may be beneficial for breaking the “plateau” of functional recovery in chronic stroke. To further assess the potential of such task-specific VR-based training (as compared to standard of care) for upper extremity rehabilitation in people with acute stroke, another study with intensive intervention (five times a week for four weeks) and fully monitoring of dose parameters is underway.
